# Risk of Shoulder Injury Related to Vaccine Administration for COVID-19 and Influenza Vaccinations

**DOI:** 10.1001/jamainternmed.2026.5899

**Published:** 2026-11-04

**Authors:** Chengyi Zheng, Lei Qian, Lina S. Sy, Gabriella V. Goodwin, Meiyu Yeh, Ronald A. Navarro, Dawn T. Asamura, Denison S. Ryan, Jonathan Duffy, Jennifer C. Nelson, Bruno J. Lewin, Hung Fu Tseng

**Affiliations:** 1 Department of Research & Evaluation, Kaiser Permanente Southern California, Pasadena; 2 Department of Orthopedic Surgery, Kaiser Permanente South Bay Medical Center, Harbor City, California; 3 Kaiser Permanente Bernard J. Tyson School of Medicine, Pasadena, California; 4 Immunization Safety Office, Centers for Disease Control and Prevention, Atlanta, Georgia; 5 Kaiser Permanente Washington Health Research Institute, Seattle; 6 Department of Family Medicine, Kaiser Permanente Los Angeles Medical Center, Los Angeles, California

## Abstract

**Importance:**

The National Academies of Sciences, Engineering, and Medicine identified a critical evidence gap regarding the risk of shoulder injury related to vaccine administration (SIRVA) after COVID-19 vaccination.

**Objective:**

To compare the risk of SIRVA after COVID-19 vs influenza vaccinations in adults.

**Design, Setting, and Participants:**

This population-based cohort study included individuals aged at least 18 years who received a COVID-19 or influenza vaccination administered via intramuscular deltoid injection between August 1, 2023, and April 30, 2024, at Kaiser Permanente Southern California. Data were analyzed from June 2024 to September 2026.

**Exposure:**

Receipt of COVID-19 vaccination or influenza vaccination.

**Main Outcomes and Measures:**

The primary outcome was incident SIRVA, defined as shoulder symptoms beginning at or before 7 days and persisting more than 30 days after vaccination, identified using a validated natural language processing algorithm. Incidences were calculated per 10 000 vaccinations. Adjusted relative risks (RRs), risk differences, and 95% CIs were estimated using robust Poisson regression with inverse probability of treatment weighting.

**Results:**

A total of 1 366 362 individuals (mean [SD] age at vaccination, 58.4 [18.2] years; 782 994 female [57.3%]) across 1 901 588 vaccinations were included (1 076 609 vaccinations [56.7%] were administered to females). Among 1 830 636 vaccinations in the primary analysis, 252 SIRVA cases were identified as follows: 110 cases (0.018%) after 596 614 COVID-19 vaccinations and 142 cases (0.012%) after 1 234 022 influenza vaccinations. An additional 13 SIRVA cases (0.018%) were identified among 70 952 simultaneous COVID-19 and influenza vaccinations. Most COVID-19 vaccines were Pfizer-BioNTech messenger RNA (667 455 of 667 566 [99.98%]), and influenza vaccines were predominantly standard-dose (866 530 of 1 304 974 [66.40%]) or high-dose (438 183 of 1 304 974 [33.58%]) inactivated quadrivalent formulations. SIRVA incidence was higher after COVID-19 than influenza vaccination, at an incidence of 1.84 (95% CI, 1.53-2.22) vs 1.15 (95% CI, 0.98-1.36) per 10 000 vaccinations, corresponding to an adjusted absolute increase of 0.67 cases (95% CI, 0.15-1.19) per 10 000 vaccinations (adjusted RR, 1.63; 95% CI, 1.16-2.28). Among adults 65 years or older, SIRVA incidence was 2.11 (95% CI, 1.67-2.67) per 10 000 for COVID-19 vaccination and 1.63 (95% CI, 1.28-2.07) per 10 000 for high-dose influenza vaccination (adjusted RR, 1.41; 95% CI, 0.92-2.15).

**Conclusions and Relevance:**

Results of this cohort study suggest that although SIRVA was rarely associated with both vaccines, COVID-19 vaccination was associated with approximately 7 additional cases per 100 000 vaccinations compared with influenza vaccination. Among adults aged 65 years or older, SIRVA risk did not differ significantly between COVID-19 and high-dose influenza vaccination. The small absolute risk of SIRVA should be considered alongside the established benefits of COVID-19 and influenza vaccination in preventing severe disease and hospitalization.

## Key Points

**Question:**

Is the risk of shoulder injury related to vaccine administration (SIRVA) higher after COVID-19 vaccination than after influenza vaccination in adults?

**Findings:**

In this population-based cohort study of more than 1.8 million vaccinations, SIRVA appeared rare but may occur more frequently after COVID-19 vaccination than influenza vaccination. Among adults 65 years or older, SIRVA risk did not differ significantly between COVID-19 and high-dose influenza vaccination.

**Meaning:**

Results suggest that vaccine-associated inflammatory responses, in addition to injection technique, may contribute to SIRVA risk, and these findings help address a key evidence gap regarding SIRVA after COVID-19 vaccination; however, the small absolute risk of SIRVA, including among older adults receiving recommended high-dose influenza vaccines, should be considered alongside the established benefits of vaccination.

## Teaser

This cohort study uses a validated natural language processing algorithm to access if the incidence of shoulder injury related to vaccine administration (SIRVA) is higher after COVID-19 vs influenza vaccination at a health care system in California.

## Introduction

Shoulder injury related to vaccine administration (SIRVA) was added to the National Vaccine Injury Compensation Program vaccine injury table in 2017, based on accumulating case series.^1^ SIRVA encompasses a spectrum of shoulder conditions after intramuscular deltoid vaccination, most likely caused by inadvertent injection into the shoulder joint structures, triggering immune-mediated inflammation and persistent symptoms.^3^ Additional potential contributors include direct mechanical trauma from needle insertion, individual susceptibility factors, or vaccine-specific inflammatory responses. The long-term prognosis of SIRVA remains poorly characterized, but recovery may be variable and prolonged, warranting larger prospective studies with longer follow-up.^3^

Population-based epidemiological studies of SIRVA have been limited by the absence of specific diagnostic codes, the condition’s rarity, and the need to distinguish true SIRVA from transient postvaccination reactogenicity.^5^ We previously developed and validated a natural language processing (NLP) algorithm for identifying SIRVA cases at Kaiser Permanente Southern California (KPSC) and identified advanced age, female sex, and specific vaccine types as potential risk factors for SIRVA.^6^

Case reports and passive surveillance studies identified SIRVA after COVID-19 vaccination^8^ but could not quantify its incidence relative to other routinely administered vaccines. The 2024 National Academy of Sciences, Engineering, and Medicine identified this as an important evidence gap, noting reliance on case reports rather than controlled epidemiological studies.^12^ As COVID-19 vaccines have transitioned to annual administration alongside influenza vaccines, both of which are delivered by intramuscular deltoid injection, understanding their comparative SIRVA risk is of direct clinical and public health relevance. We used an active-comparator cohort design and a validated NLP algorithm to compare SIRVA risk between COVID-19 and influenza vaccination, with adjustment for measured confounders.

## Methods

### Study Design and Population

This retrospective cohort study was approved by the KPSC institutional review board, with a waiver of informed consent because it was a minimal-risk observational study. The study was conducted at KPSC, an integrated health care system serving 5 million diverse members and a participating site in the Vaccine Safety Datalink (VSD), a collaboration for vaccine safety surveillance and research between the Centers for Disease Control and Prevention (CDC) and health care organizations. KPSC’s prepaid model promotes within-system care, enabling comprehensive capture of member data, including sociodemographic data, encounters, diagnoses, laboratory tests, pharmacy use, and vaccinations. The study was conducted consistent with federal law and CDC policy (45 Code of Federal Regulations [CFR] part 46.114 ^13^ and 21 CFR part 56.114 ^14^). This study followed the Strengthening the Reporting of Observational Studies in Epidemiology (STROBE) reporting guideline.

We included members 18 years and older who received a COVID-19 or influenza vaccination at a KPSC facility between August 1, 2023, and April 30, 2024. Race and ethnicity data were self-reported and recorded in the electronic health record; the categories reported were Asian or Other Pacific Islander, Black, Hispanic, White, or other race and ethnicity (including American Indian or Alaska Native, multiracial, and unknown). Race and ethnicity were included as social variables in propensity score models used to generate inverse probability of treatment weights because they may be associated with differences in health care access, vaccination patterns, and risk of SIRVA. We required continuous enrollment (allowing a 31-day gap) for 12 months before vaccination and for 180 days after vaccination to capture baseline covariates and outcome.

### Exposure, Index Date, and Outcome

The exposure was receipt of a COVID-19 vaccine (exposed) or an influenza vaccine (comparator) (eFigure 1 in Supplement 1). The vaccination date was the index date. Members could contribute multiple vaccinations, with person-level baseline characteristics assigned at each vaccination event. We excluded simultaneous COVID-19 and influenza vaccinations (same day and same arm) from the primary analysis and examined them in a secondary analysis comparing simultaneous vaccination with influenza vaccine alone. COVID-19 or influenza vaccinations coadministered with a non–COVID-19, noninfluenza vaccine in the same arm on the same day were retained. The primary outcome was SIRVA cases identified from clinical records within 180 days after vaccination. Confirmed cases required symptom onset within 7 days after vaccination and symptoms lasting more than 30 days.

### SIRVA Case Definition and Identification

The SIRVA case definition required 5 criteria as follows: (1) anatomical concordance with vaccination arm, (2) symptom onset within 7 days of vaccination, (3) symptom duration greater than 30 days to exclude transient reactogenicity events, (4) vaccination identified as a possible cause, and (5) no other identifiable causes for the shoulder condition. We identified potential cases using the *International Classification of Diseases, Tenth Revision, Clinical Modification *(*ICD-10-CM*) codes for shoulder conditions (eTables 1-5 in Supplement 1) occurring within 180 days postvaccination but not within 365 days prevaccination. Prescheduled shoulder appointments and shoulder conditions predating vaccination were excluded.^6^

The validated NLP algorithm searched clinical notes for anatomical, temporal, and causal relationships between vaccinations and shoulder symptoms and classified cases as definite, probable, or possible, based on the strength of the evidence.^7^ Based on the 95.5% confirmation rate in our prior study,^6^ NLP-classified definite cases were considered confirmed without structured medical record review. These cases were reviewed for quality assurance (C.Z.). NLP-classified probable and possible cases underwent medical record review by research associates, with 40 randomly selected cases independently reviewed by an orthopedic surgeon (R.A.N.) to assess interrater reliability (eMethods in Supplement 1). Individuals with confirmed SIRVA remained eligible to contribute subsequent vaccination events during the study period. For confirmed cases with additional same-arm vaccinations within 180 days, we manually reviewed records to verify the SIRVA-associated vaccination.

### Statistical Analysis

In primary analyses, each vaccination was the unit of analysis. In secondary analyses, we treated simultaneous same-arm, same-day vaccinations as 1 analytic unit. Subgroup analyses compared COVID-19 vs influenza vaccination within age groups (18-49, 50-64, and ≥65 years). Among adults 65 years or older, we compared COVID-19 vaccination separately with high-dose inactivated influenza vaccine quadrivalent (HD-IIV4) and standard-dose inactivated influenza vaccine quadrivalent (SD-IIV4), because these formulations differ in antigen content and immunogenicity. Adjuvanted and recombinant influenza vaccines were not excluded; however, no adjuvanted vaccines were administered, and recombinant vaccine use was minimal (approximately 0.02% of influenza vaccinations). A sensitivity analysis restricted SIRVA cases to symptom onset within 2 days of vaccination, a threshold used in prior SIRVA studies.^4^

Inverse probability of treatment weighting (IPTW) using logistic regression-derived propensity scores balanced individual-level characteristics between vaccine groups, with stabilized weights applied to reduce variability from extreme weight values and maintain the original sample size in each group. Covariate balance was assessed using absolute standardized difference (ASD), with ASD less than 0.10 considered indicative of negligible imbalance.^15^ Covariates in the propensity score model included demographics, comorbidities, health care utilization, vaccination history, and established risk factors for shoulder conditions (eTable 6 in Supplement 1).^16^ Unadjusted and IPTW-adjusted relative risks (RRs) with 95% CIs were estimated using Poisson regression with robust sandwich variance estimators to account for within-person clustering.^29^ Corresponding risk differences (RDs) with 95% CIs were derived from model-based incidence estimates and expressed per 10 000 vaccinations.

Two covariates—total prior vaccinations in the 6 months before the index date and the month of vaccination—were excluded from the propensity score model because extreme imbalance (ASD >1.0) reflected structural differences in vaccine availability and seasonal uptake patterns rather than patient characteristics. We instead adjusted for these covariates directly in the Poisson regression outcome model to preserve confounding control while avoiding propensity score instability and extreme weights. In the primary analysis, we modeled these 2 variables categorically. In the age-stratified analysis, we modeled them categorically for adults 65 years or older and as binary indicators for adults aged 18 to 49 years and 50 to 64 years. In a separate analysis restricted to adults aged 65 years or older, we modeled both variables as binary indicators, because case counts were sparse. Data were analyzed from June 2024 to September 2026, and all statistical analyses were performed using SAS, version 9.4 (SAS Institute Inc).

## Results

### Study Population and SIRVA Case Identification

After applying the eligibility criteria, a total of 1 366 362 individuals (mean [SD] age at vaccination, 58.4 [18.2] years; 782 994 female [57.3%] and 583 368 male [42.7%]) across 1 901 588 vaccinations were included (1 076 609 vaccinations [56.7%] were administered to females and 824 979 [43.3%] to males) (Figure). Among eligible vaccinations, 313 571 (16.5%) were received by Asian or Other Pacific Islander individuals, 134 951 (7.1%) by Black individuals, 704 502 (37.0%) by Hispanic individuals, 667 597 (35.1%) by White individuals, and 80 967 (4.3%) by individuals of multiple, other, or unknown race and ethnicity. Of the eligible vaccinations, 70 952 (3.7%) were simultaneous COVID-19 and influenza vaccinations and were excluded from the primary analysis, leaving a total of 1 830 636 vaccinations, 1 234 022 influenza (64.9%) and 596 614 COVID-19 (31.4%) vaccinations. Almost all COVID-19 vaccines were Pfizer-BioNTech messenger RNA (mRNA; 667 455 of 667 566 [99.98%]); nearly all influenza vaccines were SD-IIV4 (866 530 of 1 304 974[66.40%]) or HD-7 IIV4 (438 183 of 1 304 974 [33.58%]) (eTable 7 in Supplement 1).

**Figure.**
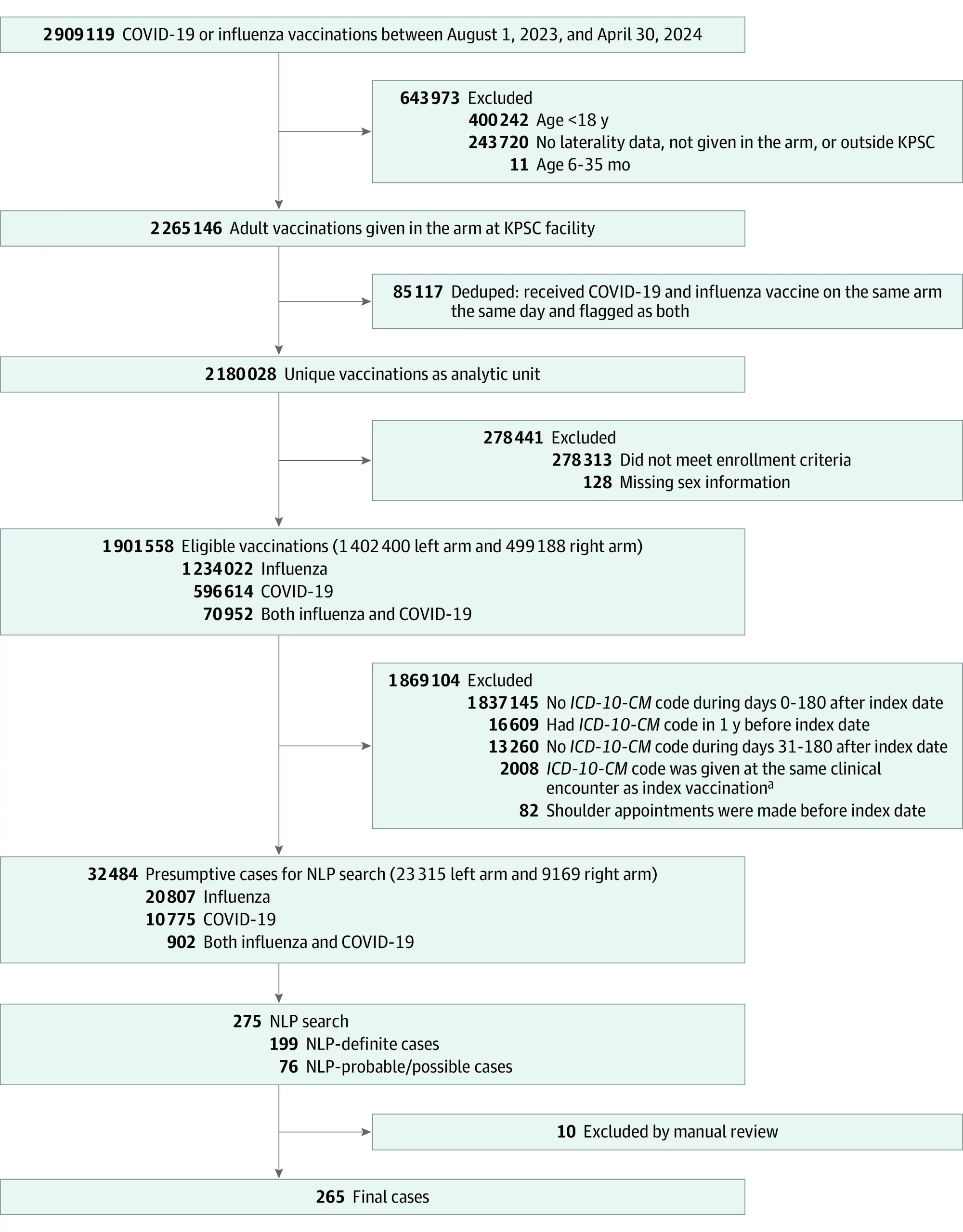
Flow Diagram Showing Cohort Eligibility, Case Finding by the Natural Language Processing Algorithm, and Case Confirmation *ICD-10-CM* indicates *International Classification of Diseases, Tenth Revision, Clinical Modification*; KPSC, Kaiser Permanente Southern California; NLP, natural language processing. ^a^The laterality of the *ICD-10-CM* code must match the laterality of the index vaccination.

We used a previously developed and validated NLP algorithm for identifying SIRVA cases at KPSC (which had estimated an incidence of 1.22 per 10 000 vaccinations in adults in our prior study).^6^ Among 32 484 presumptive cases identified using *ICD-10-CM* codes, the NLP algorithm identified 199 definite (0.61%) and 76 probable or possible (0.23%) SIRVA cases. Manual review confirmed 66 of the latter (86.8%), yielding 265 confirmed SIRVA cases (0.82%), including 13 after simultaneous COVID-19 and influenza vaccination (0.04%) which were excluded from the primary analysis. In total, 252 SIRVA cases were identified—110 cases (0.34%) after COVID-19 vaccinations and 142 cases (0.44%) after influenza vaccinations. Agreement for 40 independently reviewed cases was 85.0% (Cohen κ = 0.42; 95% CI, 0-0.85) (eTable 8 in Supplement 1). The term *SIRVA* was explicitly documented in 7 confirmed SIRVA cases (2.6%).

Among confirmed SIRVA cases, the median time to the first shoulder-related encounter was 46.0 days after influenza vaccination (range, 0-174 days) and 51.5 days after COVID-19 vaccination (range, 1-169 days); median times to the last shoulder-related encounter were 99.5 (range, 31-178) days and 130.5 (range, 41-179) days, respectively (eTable 9 in Supplement 1). Additionally, 81 patients (30.6%) had at least 1 additional vaccination administered in the same arm within 180 days before or after the SIRVA-associated vaccination (eTable 10 in Supplement 1). Manual chart review confirmed correct identification of the SIRVA-associated vaccination in all 81 cases.

### Descriptive Analysis and IPTW

Individuals receiving COVID-19 vaccination were older than those receiving influenza vaccination (mean [SD] age, 62.6 [17.1] vs 56.6 [18.3] years), with a higher proportion 65 years or older (331 250 [55.5%] vs 482 915 [39.1%]) (Table 1). COVID-19 vaccine recipients were slightly more likely to be White (239 440 [40.1%] vs 401 555 [32.5%]) and less likely to be Hispanic (185 721 [31.1%] vs 495 460 [40.2%]). COVID-19 vaccine recipients had greater comorbidity burden, longer health plan membership, and greater prior health care use. After IPTW, all covariates included in the propensity score model achieved balance, with no covariate retaining an ASD greater than 0.10 (eFigure 2 and eTable 11 in Supplement 1).

**Table 1.** Characteristics of Cohorts Who Received COVID-19 and Influenza Vaccination Before Weighting, Kaiser Permanente Southern California, August 1, 2023, to April 30, 2024

Characteristic	Vaccination, No. (%)^a^		ASD^b^
	Influenza (n = 1 234 022)	COVID-19 (n = 596 614)	
Age at index date, y			
Mean (SD)	56.6 (18.3)	62.6 (17.1)	0.34
Median (IQR)	59 (43-71)	67 (52-75)	
18-49	424 191 (34.4)	130 617 (21.9)	0.35
50-64	326 916 (26.5)	134 747 (22.6)	
≥65	482 915 (39.1)	331 250 (55.5)	
Sex			
Female	712 854 (57.8)	327 818 (54.9)	0.06
Male	521 168 (42.2)	268 796 (45.1)	
Race and ethnicity^c^			
Asian and Other Pacific Islander	200 967 (16.3)	100 987 (16.9)	0.20
Black	83 730 (6.8)	46 232 (7.7)	
Hispanic	495 460 (40.2)	185 721 (31.1)	
White	401 555 (32.5)	239 440 (40.1)	
Multiple, other, and unknown^d^	52 310 (4.2)	24 234 (4.1)	
Body mass index^e^			
Mean (SD)	29.5 (6.8)	29.0 (6.5)	0.07
Median (IQR)	28.4 (24.7-33.0)	27.9 (24.4-32.3)	
<25	296 248 (24.0)	158 562 (26.6)	0.08
25-<30	371 789 (30.1)	186 668 (31.3)	
30-<35	246 178 (19.9)	114 072 (19.1)	
≥35	197 378 (16.0)	85 647 (14.4)	
Unknown	122 429 (9.9)	51 665 (8.7)	
Alcohol history^f^	303 452 (24.6)	169 134 (28.3)	0.09
Smoking history^f^	302 525 (24.5)	166 535 (27.9)	0.08
Length of membership, y			
1-5	362 039 (29.3)	142 576 (23.9)	0.16
6-10	279 681 (22.7)	122 610 (20.6)	
≥11	592 302 (48.0)	331 428 (55.6)	
Prior outpatient or virtual visits^f^			
0	26 530 (2.1)	897 (0.2)	0.27
1-4	363 992 (29.5)	133 405 (22.4)	
5-10	381 302 (30.9)	190 794 (32.0)	
≥11	462 198 (37.5)	271 518 (45.5)	
Prior ED visits^f^			
0	970 111 (78.6)	472 465 (79.2)	0.01
1	168 511 (13.7)	79 508 (13.3)	
≥2	95 400 (7.7)	44 641 (7.5)	
Prior inpatient visits^f^			
0	1 134 758 (92.0)	547 552 (91.8)	0.008
1	72 172 (5.8)	35 275 (5.9)	
≥2	27 092 (2.2)	13 787 (2.3)	
Total prior COVID-19 vaccinations			
0-1	57 125 (4.6)	4466 (0.7)	0.72
2-3	529 214 (42.9)	104 655 (17.5)	
4-5	602 355 (48.8)	402 634 (67.5)	
≥6	45 328 (3.7)	84 859 (14.2)	
Total prior vaccinations, No. (%)^g^			
0	980 322 (79.4)	142 413 (23.9)	1.34
1	186 679 (15.1)	321 784 (53.9)	
2-3	62 983 (5.1)	123 836 (20.8)	
≥4	4038 (0.3)	8581 (1.4)	
Month of vaccination			
August-October	876 355 (71.0)	152 802 (25.6)	1.04
November-January	295 763 (24.0)	308 502 (51.7)	
February-April	61 904 (5.0)	135 310 (22.7)	
Vaccinator credential			
Licensed vocational nurse	961 836 (77.9)	501 650 (84.1)	0.18
Medical assistant	149 901 (12.1)	45 898 (7.7)	
Registered nurse	110 044 (8.9)	41 740 (7.0)	
Student	7418 (0.6)	6062 (1.0)	
Other credentials	4823 (0.4)	1264 (0.2)	
Vaccination arm (left)	901 825 (73.1)	443 301 (74.3)	0.03
Simultaneously administered with non–COVID-19, noninfluenza vaccination, No. (%)^h^	44 095 (3.6)	15 603 (2.6)	0.06
Charlson Comorbidity Index score^f^			
0	662 645 (53.7)	270 877 (45.4)	0.17
1	174 113 (14.1)	91 292 (15.3)	
≥2	397 264 (32.2)	234 445 (39.3)	
Comorbidities^i^			
Anemia	174 650 (14.2)	94 702 (15.9)	0.05
Angina	280 086 (22.7)	136 831 (22.9)	0.006
Arthropathy	395 402 (32.0)	237 642 (39.8)	0.16
Cervical radiculopathy	57 654 (4.7)	30 466 (5.1)	0.02
Depression	245 577 (19.9)	124 562 (20.9)	0.02
Diabetes	291 877 (23.7)	161 180 (27.0)	0.08
Fibromyalgia	20 278 (1.6)	10 003 (1.7)	0.003
Immune-mediated gastrointestinal disorders	13 099 (1.1)	7414 (1.2)	0.02
Hyperlipidemia	695 254 (56.3)	391 231 (65.6)	0.19
Hypertension	538 687 (43.7)	310 572 (52.1)	0.17
Immunosuppression	230 830 (18.7)	134 829 (22.6)	0.10
Thyroid disorders	180 282 (14.6)	102 021 (17.1)	0.07
Myocardial infarction	45 243 (3.7)	26 699 (4.5)	0.04
Muscle disorders	119 077 (9.6)	60 925 (10.2)	0.02
Osteoporosis	123 147 (10.0)	83 490 (14.0)	0.12
Sleep disorders	190 510 (15.4)	110 722 (18.6)	0.08
Stroke	66 471 (5.4)	39 915 (6.7)	0.06
Neighborhood Deprivation Index, quartile^j^			
First	344 693 (27.9)	202 137 (33.9)	0.16
Second	343 253 (27.8)	170 301 (28.5)	
Third	301 145 (24.4)	130 422 (21.9)	
Fourth	244 479 (19.8)	93 567 (15.7)	
Unknown	452 (0)	187 (0)	
Neighborhood-level education			
<50% With a high school education	59 088 (4.8)	20 864 (3.5)	0.07
≥50% With a high school education	1 174 894 (95.2)	575 743 (96.5)	
Unknown	40 (0)	7 (0)	

Abbreviations: ASD, absolute standardized difference; ED, emergency department.

^a^ The unit of analysis is vaccination. Characteristics are presented for individuals at the time of each vaccination. Because individuals could receive multiple vaccinations during the study period, the same person may be represented in both vaccine groups. The numbers of unique individuals in the influenza and COVID-19 vaccination groups were 1 231 959 and 544 577, respectively. The number of individuals who received both influenza and COVID-19 vaccinations was 477 390.

^b^ ASD values greater than or equal to 0.10 indicate meaningful imbalance between groups.

^c^ Race and ethnicity data were self-reported and recorded in the electronic health record. All races listed are non-Hispanic.

^d^ Multiple included individuals who self-reported 2 or more races; other included American Indian or Alaska Native individuals and those who did not declare a race; and unknown included individuals with missing race or ethnicity data.

^e^ The most recent data in the year before the index vaccination. BMI is calculated as weight in kilograms divided by height in meters squared.

^f^ Twelve months before the index vaccination.

^g^ Six months before the index vaccination.

^h^ COVID-19 or influenza vaccination coadministered with another vaccine that was neither COVID-19 nor influenza in the same arm on the same day.

^i^ Lookback periods are defined in eTable 6 in Supplement 1.

^j^ The Neighborhood Deprivation Index is a composite measure of socioeconomic status at the census tract level, categorized into quartiles (1st-4th), with the first representing the least deprived and the fourth the most deprived. Quartiles were defined based on the 2022 Kaiser Permanente Southern California enrolled population.

### SIRVA Risk in the Overall Cohort

SIRVA incidence was 1.84 (95% CI, 1.53-2.22) per 10 000 COVID-19 vaccinations and 1.15 (95% CI, 0.98-1.36) per 10 000 influenza vaccinations. The adjusted RR was 1.63 (95% CI, 1.16-2.28), corresponding to an absolute adjusted RD of 0.67 (95% CI, 0.15-1.19) additional SIRVA cases per 10 000 vaccinations (Table 2). Simultaneous administration of COVID-19 and influenza vaccines was not associated with a statistically significant difference in SIRVA risk compared with influenza vaccination alone (adjusted RR, 1.97; 95% CI, 0.82-4.75; absolute adjusted RD, 0.73; 95% CI, −0.64 to 2.10 cases per 10 000 vaccinations).

**Table 2.** SIRVA Incidence, Relative Risks, and Absolute Risk Differences After COVID-19 Vaccination Compared With Influenza Vaccination, Kaiser Permanente Southern California, August 1, 2023, to April 30, 2024

Vaccination	Vaccinations, No.	Cases, No.	Incidence (95% CI)^a^	Relative risk (95% CI)		Absolute risk difference per 10 000 (95% CI)	
				Unadjusted	IPTW adjusted^b^	Unadjusted	IPTW adjusted^b^
Influenza	1 234 022	142	1.15 (0.98 to 1.36)	1 [Reference]	1 [Reference]	1 [Reference]	1 [Reference]
COVID-19	596 614	110	1.84 (1.53 to 2.22)	1.60 (1.25 to 2.05)	1.63 (1.16 to 2.28)	0.69 (0.30 to 1.09)	0.67 (0.15 to 1.19)
Simultaneous^c^	70 952	13	1.83 (1.06 to 3.16)	1.59 (0.90 to 2.81)	1.97 (0.82 to 4.75)	0.68 (−0.33 to 1.70)	0.73 (−0.64 to 2.10)

Abbreviations: IPTW, inverse probability of treatment weighting; NA, not applicable; SIRVA, shoulder injury related to vaccine administration.

^a^ Incidence (95% CI): SIRVA per 10 000 vaccinations.

^b^ Total prior vaccinations in the 6 months before the index date and month of vaccination were removed from IPTW analysis but directly adjusted in the Poisson regression model as categorical variables.

^c^ COVID-19 vaccine and influenza vaccine were coadministered in the same arm on the same day.

### Age-Specific SIRVA Risk

For COVID-19 vaccinations, incidences were 1.00 (95% CI, 0.58-1.71), 2.00 (95% CI, 1.37-2.92), and 2.11 (95% CI, 1.67-2.67) per 10 000 vaccinations in adults aged 18 to 49 years, 50 to 64 years, and 65 years or older, respectively (Table 3). Corresponding rates for influenza vaccination were 0.71 (95% CI, 0.49-1.01), 1.07 (95% CI, 0.77-1.49), and 1.59 (95% CI, 1.28-1.99) per 10 000 vaccinations.

**Table 3.** SIRVA Incidence, Relative Risks, and Absolute Risk Differences After COVID-19 Vaccination Compared With Influenza Vaccination by Age Group, Kaiser Permanente Southern California, August 1, 2023, to April 30, 2024

Age, y^a^	Vaccination	Vaccinations, No.	Cases, No.	Incidence (95% CI)^b^	Relative risk (95% CI)		Absolute risk difference per 10 000 (95% CI)	
					Unadjusted	IPTW adjusted^c^	Unadjusted	IPTW adjusted^c^
18-49	Influenza	424 191	30	0.71 (0.49 to 1.01)	1 [Reference]	1 [Reference]	1 [Reference]	1 [Reference]
	COVID-19	130 617	13	1.00 (0.58 to 1.71)	1.41 (0.73 to 2.70)	1.44 (0.70 to 2.93)	0.29 (−0.31 to 0.89)	0.33 (−0.38 to 1.04)
50-64	Influenza	326 916	35	1.07 (0.77 to 1.49)	1 [Reference]	1 [Reference]	1 [Reference]	1 [Reference]
	COVID-19	134 747	27	2.00 (1.37 to 2.92)	1.87 (1.13 to 3.09)	1.84 (0.99 to 3.45)	0.93 (0.10 to 1.77)	0.96 (−0.12 to 2.05)
≥65	Influenza	482 915	77	1.59 (1.28 to 1.99)	1 [Reference]	1 [Reference]	1 [Reference]	1 [Reference]
	COVID-19	331 250	70	2.11 (1.67 to 2.67)	1.33 (0.96 to 1.83)	1.53 (0.97 to 2.39)	0.52 (−0.09 to 1.13)	0.75 (−0.02 to 1.53)

Abbreviations: IPTW, inverse probability of treatment weighting; SIRVA, shoulder injury related to vaccine administration.

^a^ Age in years at the index vaccination.

^b^ Incidence (95% CI): SIRVA per 10 000 vaccinations.

^c^ Total prior vaccinations in the 6 months before the index date and month of vaccination were removed from IPTW analysis but directly adjusted in Poisson regression model as categorical variables for age 65 years or older estimates and as binary variables (yes or no for total prior vaccination and winter vs nonwinter months for month of vaccination) for age 18 to 49 years and 50 to 64 years estimates. The winter months were defined as November to January.

In age-stratified subgroup analyses, point estimates were directionally consistent with the primary result, although no individual stratum reached statistical significance. Among adults aged 18 to 49 years, 50 to 64 years, and 65 years or older, adjusted RRs for COVID-19 vs influenza vaccination were 1.44 (95% CI, 0.70-2.93), 1.84 (95% CI, 0.99-3.45), and 1.53 (95% CI, 0.97-2.39), respectively. The corresponding absolute adjusted RDs were 0.33 (95% CI, −0.38 to 1.04), 0.96 (95% CI, −0.12 to 2.05), and 0.75 (95% CI, −0.02 to 1.53) SIRVA cases per 10 000 vaccinations.

### SIRVA Risk by Influenza Vaccine Formulation in Adults 65 Years or Older

Among adults aged 65 years or older who received an influenza vaccine, 417 398 received HD-IIV4 (86.4%) and 65 517 received SD-IIV4 (13.6%). SIRVA incidence after HD-IIV4 and SD-IIV4 was 1.63 (95% CI, 1.28-2.07) and 1.37 (95% CI, 0.71-2.64) per 10 000 vaccinations, respectively (Table 4). Compared with SD-IIV4, COVID-19 vaccination was associated with significantly higher SIRVA risk (adjusted RR, 2.54; 95% CI, 1.18-5.47). In contrast, comparison of COVID-19 vaccination with HD-IIV4 (a recommended influenza vaccine for this age group) showed no statistically significant difference in SIRVA risk (adjusted RR, 1.41; 95% CI, 0.92-2.15). The absolute risk difference between COVID-19 vaccination and HD-IIV4 was 0.64 (95% CI, −0.20 to 1.44) per 10 000 vaccinations.

**Table 4.** SIRVA Incidence, Relative Risks, and Absolute Risk Differences After COVID-19 Vaccination Compared With HD-IIV4 and SD-IIV4 Influenza Vaccination Among Individuals 65 Years or Older, Kaiser Permanente Southern California, August 1, 2023, to April 30, 2024

Vaccination	Vaccinations, No.	Cases, No.	Incidence (95% CI)^a^	Relative risk (95% CI)		Absolute risk difference per 10 000 (95% CI)	
				Unadjusted	IPTW adjusted^b^	Unadjusted	IPTW adjusted^b^
HD-IIV4	417 398	68	1.63 (1.28 to 2.07)	1 [Reference]	1 [Reference]	1 [Reference]	1 [Reference]
COVID-19	331 250	70	2.11 (1.67 to 2.67)	1.30 (0.93 to 1.80)	1.41 (0.92 to 2.15)	0.48 (−0.14 to 1.11)	0.64 (−0.20 to 1.44)
SD-IIV4	65 517	9	1.37 (0.71 to 2.64)	1 [Reference]	1 [Reference]	1 [Reference]	1 [Reference]
COVID-19	331 250	70	2.11 (1.67 to 2.67)	1.54 (0.77 to 3.08)	2.54 (1.18 to 5.47)	0.74 (−0.29 to 1.76)	1.50 (0.56 to 2.43)

Abbreviations: HD-IIV4, high-dose inactivated influenza vaccine quadrivalent; SD-IIV4, standard-dose inactivated influenza vaccine quadrivalent; IPTW, inverse probability of treatment weighting; SIRVA, shoulder injury related to vaccine administration.

^a^ Incidence (95% CI): SIRVA per 10 000 vaccinations.

^b^ In addition to IPTW adjustment, the model was further adjusted for total vaccines received in the 6 months before the index date (yes or no) and month of vaccination (winter vs nonwinter months).

### Sensitivity Analysis

Among the 265 SIRVA cases, 251 had symptom onset within 2 days after vaccination (94.7%) and 14 within days 3 to 7 (5.3%). After excluding 13 simultaneous vaccination cases and restricting to onset within 2 days, the adjusted RR slightly increased to 1.72 (95% CI, 1.22-2.44) (eTable 12 in Supplement 1).

## Discussion

SIRVA was rarely associated with both COVID-19 and influenza vaccinations, with incidences of 1.84 and 1.15 per 10 000 vaccinations, respectively (adjusted absolute difference, 0.67 per 10 000).^30^ Despite the higher incidence after COVID-19 vaccination, the benefits of COVID-19 and influenza vaccination in preventing severe disease and hospitalization may outweigh the small absolute and relative risks of SIRVA.

Age-stratified RR point estimates remained consistently above 1.0 but were not statistically significant, likely reflecting low statistical power. Simultaneous administration of COVID-19 and influenza vaccines also produced an elevated but nonsignificant estimate (RR, 1.97; 95% CI, 0.82-4.75).

Among adults 65 years or older, COVID-19 vaccination was associated with a statistically significantly higher SIRVA risk than SD-IIV4 vaccination. Notably, this comparison was based on a small number of events—only 9 SIRVA cases among 65 517 recipients of standard-dose influenza vaccine, limiting precision. Interpretation of this finding must also consider influenza vaccination practice. In this cohort, 86.4% of influenza vaccine recipients aged 65 years or older received HD-IIV4, consistent with clinical guidelines that recommend enhanced influenza vaccines, such as HD-IIV4, for older adults.^31^ When COVID-19 vaccination was compared with HD-IIV4 vaccination, no statistically significant difference in SIRVA risk was observed.

A potential dose-response pattern across vaccine formulations may provide insight into SIRVA pathophysiology. SIRVA incidence after HD-IIV4 (1.63 per 10 000 vaccinations) fell between SD-IIV4 (1.37 per 10 000) and COVID-19 vaccination (2.11 per 10 000), although SD-IIV4 estimates in adults 65 years or older were based on few cases and should be interpreted cautiously. This gradient aligns with known differences in vaccine reactogenicity: high-dose influenza vaccines contain 4 times the antigen of standard-dose formulations^32^ and may elicit greater local inflammatory responses,^33^ whereas COVID-19 mRNA vaccines may elicit stronger inflammatory responses than inactivated influenza vaccines, including HD-IIV4,^34^ likely because lipid nanoparticles may trigger robust innate immune responses.^37^ Consistent with a possible dose-response association, our prior study^6^ also suggested a higher risk of SIRVA following high-dose compared with standard-dose influenza vaccination. A purely mechanical mechanism would not fully explain differences across vaccine formulations if administration practices were comparable. Together, these findings may suggest that immune-mediated inflammatory responses contribute to SIRVA pathophysiology in addition to injection technique, although this hypothesis warrants further investigation. Comparative studies of other reactogenic vaccines, including recombinant zoster vaccine, may help evaluate whether vaccine-specific inflammatory responses contribute to SIRVA risk.

Paradoxically, an increase in SIRVA incidence was associated with age in this cohort, despite the well-established age-related decline in vaccine reactogenicity associated with immunosenescence.^39^ This pattern may reflect age-related anatomical changes that independently increase SIRVA susceptibility through mechanisms that outweigh the declining systemic reactogenicity. First, sarcopenia and progressive fatty infiltration of deltoid muscle, which are common after approximately 40 years of age,^40^ may reduce effective target-tissue depth, increasing the probability of needle overpenetration. Second, age-related reduction in rotator cuff vascularity after age 40 years may impair local clearance of inadvertently deposited vaccine antigen.^44^ Once vaccine components enter shoulder structures, degenerative shoulder changes prevalent in aging populations may further amplify the local inflammatory response.^45^ The susceptibility may be compounded by inflammaging, a chronic, low-grade systemic inflammatory state associated with aging.^46^

Although this study did not measure injection site, deltoid muscle depth, or other aspects of vaccine administration technique, adherence to recommended intramuscular injection practices remains an important component of SIRVA prevention.^7^ These considerations may be particularly relevant in older adults because of age-related anatomical changes. The term *SIRVA* was explicitly documented in only 7 of 265 confirmed cases (2.6%), consistent with our prior study.^6^ The infrequent documentation underscores the need for greater clinician and patient awareness to facilitate timely evaluation and management. Continued competency-based training in anatomical landmarking and individualized needle selection may reinforce recommended administration practices.^49^

Past SIRVA studies have varied in their symptom-onset requirements, with 2-day and 7-day onset windows most commonly used.^3^ We applied a 7-day onset window to accommodate later onset, with only 5% of SIRVA cases occurring on days 3 to 7, consistent with our prior study.^6^ The sensitivity analysis using the narrower 2-day onset window did not meaningfully alter the COVID-19 vaccine risk estimate.

### Strengths and Limitations

This study has some strengths. The validated NLP algorithm enabled systematic ascertainment of this rare adverse event. The active-comparator design, using influenza vaccination rather than an unvaccinated reference group, minimizes confounding by indication and healthy-vaccinee bias. The contemporaneous exposure period controls for secular trends in health care use and documentation practices. IPTW-based confounding adjustment reduced confounding by measured confounders.

This study also has some limitations. The NLP algorithm was developed and validated at KPSC; application to other VSD sites would require site-specific development and validation, which was beyond this study’s scope. Although KPSC serves 5 million sociodemographically diverse members, the single-system design may limit generalizability.

Case ascertainment relied on care seeking and clinical documentation of medically attended SIRVA and may therefore underestimate true SIRVA incidence. Differential awareness, care seeking, or documentation after COVID-19 vs influenza vaccination may also have affected the RR estimate.

The observational nature of this study limits causal inference. COVID-19 vaccine recipients differed clinically from influenza vaccine recipients, and residual confounding from unmeasured variables may persist despite IPTW adjustment. These variables include needle length, injection site within the deltoid, or undiagnosed preexisting shoulder conditions. Although we adjusted for vaccinator credential, it does not directly capture experience or technique.

Not all definite cases underwent formal medical record confirmation because of the intensive longitudinal review required and the NLP algorithm’s high prior confirmation rate. Agreement was high among double-reviewed probable and possible cases, although the small sample and predominance of positive cases yielded a wide CI for Cohen κ. Residual outcome misclassification cannot be excluded.

The study period comprised predominantly Pfizer-BioNTech mRNA COVID-19 doses, limiting generalizability to other COVID-19 vaccine platforms that may differ in immunogenicity. Among adults aged 65 years or older, only 13.6% received SD-IIV4, resulting in wide CIs despite statistical significance. Finally, analyses were restricted to the 2023 to 2024 season and did not capture earlier COVID-19 vaccination periods, when formulations and administration practices may have differed. Whether these risk patterns remain consistent as vaccine formulations and administration protocols evolve warrants further investigation.

## Conclusions

Results of this population-based cohort study suggest that SIRVA was rare after both COVID-19 and influenza vaccination. COVID-19 vaccination was associated with approximately 7 additional cases per 100 000 vaccinations compared with influenza vaccination. Among adults 65 years or older, COVID-19 vaccination was not associated with a significantly different SIRVA risk compared with high-dose influenza vaccination, a recommended formulation. SIRVA incidence was associated with an increase across standard-dose influenza, high-dose influenza, and COVID-19 vaccines; however, estimates for standard-dose influenza in older adults may be imprecise because of few cases. This pattern may suggest that immune-mediated inflammatory responses contribute to SIRVA pathophysiology in addition to injection technique, although this interpretation is hypothesis-generating. The benefits of COVID-19 and influenza vaccination in preventing severe disease and hospitalization may outweigh the small absolute risk of SIRVA.

## Notes

Supplement 1. **eMethods. **Chart Abstraction Guidelines and Abstraction Form **eFigure 1. **Illustrations of Examples of Influenza and COVID-19 Vaccinations Administered Between August 1, 2023, and April 30, 2024 **eFigure 2. **Comparison of Absolute Standardized Difference Before and After IPTW Among Influenza-Vaccinated and COVID-19-Vaccinated Individuals, Kaiser Permanente Southern California, August 1, 2023, to April 30, 2024 **eTable 1. ***ICD-10-CM* Code Groups for Identifying Presumptive Shoulder Injury Cases **eTable 2. ***ICD-10-CM* Code Group A (Shoulder Disorder Codes From SIRVA Literature) **eTable 3. ***ICD-10-CM* Code Group B (Shoulder Disorder Codes not Previously Reported in the SIRVA Literature) **eTable 4. ***ICD-10-CM* Code Group C (Shoulder Symptom Codes) **eTable 5. ***ICD-10-CM* Code Group D (From *ICD-10-CM* Chapter 19: Injury, Poisoning and Certain Other Consequences of External Causes) **eTable 6. **Assessment Interval Prior to the Index Date for Comorbidities **eTable 7. **Types of Influenza and COVID-19 Vaccines in the Eligible, Presumptive, and SIRVA Cohorts, Kaiser Permanente Southern California, August 1, 2023, to April 30, 2024 **eTable 8. **Agreement Between the Orthopedic Surgeon and Research Associates and Adjudicated Final Classification Among 40 Probable or Possible Cases **eTable 9. **Timing of First and Last Shoulder-Related Encounters During the 180-Day Follow-Up Among Confirmed SIRVA Cases, by Vaccination Group **eTable 10. **Timing of Additional Same-Arm Vaccinations Before or After the SIRVA-Associated Vaccination Among Confirmed SIRVA Cases **eTable 11. **Characteristics of Influenza-Vaccinated and COVID-19-Vaccinated Cohorts After Weighting, Kaiser Permanente Southern California, August 1, 2023, to April 30, 2024 **eTable 12. **SIRVA Incidence and Relative Risk After COVID-19 Vaccination Compared to Influenza Vaccination, With Onset Date Within 2 Days After Vaccination, Kaiser Permanente Southern California, August 1, 2023, to April 30, 2024

Supplement 2.
Data Sharing Statement.
