# Supplementary material for: Risk of Shoulder Injury Related to Vaccine Administration for COVID-19 and Influenza Vaccinations
Source: JAMA Intern Med. 2026 Nov 4:e265899. Online ahead of print. doi: 10.1001/jamainternmed.2026.5899 (PMC13735196; doi:10.1001/jamainternmed.2026.5899)
Supplement: Supplementary file 1 [file jamainternmed-e265899-s001.pdf]

Supplemental Online Content

Zheng C, Qian L, Sy L, et al. Risk of shoulder injury related to vaccine administration for COVID-19 and influenza vaccinations. *JAMA Intern Med*. Published online November 4, 2026. doi:10.1001/jamainternmed.2026.5899

eMethods. Chart Abstraction Guidelines and Abstraction Form

eFigure 1. Illustrations of Examples of Influenza and COVID-19 Vaccinations Administered Between August 1, 2023, and April 30, 2024

eFigure 2. Comparison of Absolute Standardized Difference Before and After IPTW Among Influenza-Vaccinated and COVID-19-Vaccinated Individuals, Kaiser Permanente Southern California, August 1, 2023, to April 30, 2024

eTable 1. *ICD-10-CM* Code Groups for Identifying Presumptive Shoulder Injury Cases

eTable 2. *ICD-10-CM* Code Group A (Shoulder Disorder Codes From SIRVA Literature)

eTable 3. *ICD-10-CM* Code Group B (Shoulder Disorder Codes not Previously Reported in the SIRVA Literature)

eTable 4. *ICD-10-CM* Code Group C (Shoulder Symptom Codes)

eTable 5. *ICD-10-CM* Code Group D (From ICD-10-CM Chapter 19: Injury, Poisoning and Certain Other Consequences of External Causes)

eTable 6. Assessment Interval Prior to the Index Date for Comorbidities

eTable 7. Types of Influenza and COVID-19 Vaccines in the Eligible, Presumptive, and SIRVA Cohorts, Kaiser Permanente Southern California, August 1, 2023, to April 30, 2024

eTable 8. Agreement Between the Orthopedic Surgeon and Research Associates and Adjudicated Final Classification Among 40 Probable or Possible Cases

eTable 9. Timing of First and Last Shoulder-Related Encounters During the 180-Day Follow-Up Among Confirmed SIRVA Cases, by Vaccination Group

eTable 10. Timing of Additional Same-Arm Vaccinations Before or After the SIRVA-Associated Vaccination Among Confirmed SIRVA Cases

eTable 11. Characteristics of Influenza-Vaccinated and COVID-19-Vaccinated Cohorts After Weighting, Kaiser Permanente Southern California, August 1, 2023, to April 30, 2024

© 2026 Zheng C et al. *JAMA Internal Medicine*.

eTable 12. SIRVA Incidence and Relative Risk After COVID-19 Vaccination Compared to Influenza Vaccination, With Onset Date Within 2 Days After Vaccination, Kaiser Permanente Southern California, August 1, 2023, to April 30, 2024

This supplemental material has been provided by the authors to give readers additional information about their work.

© 2026 Zheng C et al. *JAMA Internal Medicine*.

eMethods. Chart Abstraction Guidelines and Abstraction Form

Case definition:

A case is defined as a new incident of shoulder condition that manifested in the same arm where the vaccine was administered, with onset within the first seven days of vaccination and lasting more than 30 days. Clinical records through day 180 after vaccination were reviewed to establish symptom persistence beyond 30 days and confirm the case definition. Vaccination should also be identified as a possible cause of the shoulder condition, with no other known causes.

Interested vaccination:

COVID-19 or influenza vaccine injected in an arm at a KPSC facility between August 1, 2023, and April 30, 2024.

HealthConnect review:

Review the relevant materials in the review window. Please note that patients' messages, telephone calls, etc., often include information about their shoulder conditions. The above clinical documents are often found in the 'Notes' tab of patients' charts.

Case ascertainment:

A member could receive multiple vaccinations on the same arm during the review window (-7 to 180 days). Please verify that the shoulder symptom is related to the pre-populated vaccination in section B.

© 2026 Zheng C et al. *JAMA Internal Medicine*.

Abstraction form

1. SCK Study ID (pre-populated)

2. VSD Study ID (pre-populated)

A. BACKGROUND INFORMATION

3. Abstractor initials

4. Abstraction date

B. VACCINATION

The vaccination information is pre-populated.

The member could receive vaccines on the same day at different arm but only one laterality (either left or right) is pre-populated and will be our 'interested laterality'.

5. Vaccination date (pre-populated)

5-1). Vaccine type (pre-populated) and laterality

5-2). Vaccine type (pre-populated) and laterality

5-3). Vaccine type (pre-populated) and laterality

Review the medical records on the date of the vaccination

Note any discrepancy between the clinical notes/vaccination registry with the populated data above.

If the side from the chart/registry (right, left, or both) does not match the pre-populated value above, use the chart value. If no chart value is available, then use the pre-populated value. Hereafter, this will be referred to as the "shoulder of interest" and will be populated with questions about the side of symptoms/injury.

6. Were any problems with the vaccine injection noted (e.g., administration error)?
   - ○ Yes
   - ○ No

6-1). If yes, specify errors noted.

7. Did the patient have any post-vaccination shoulder or arm (same as vaccinated) complaints noted at this visit?
   - ○ Yes
   - ○ No

8. Does the note mention any previous or pre-existing shoulder symptoms on the interested laterality?
   - Yes

© 2026 Zheng C et al. *JAMA Internal Medicine*.

◯
◯ No

8-1). Please copy and paste the relevant statement for previous shoulder symptoms and side mentioned from the chart notes.

C. PRE-EXISTING SHOULDER CONDITIONS DOCUMENTED PRIOR TO VACCINATION DATE

Working backward from the date of vaccination (including the date of vaccination) to 12 months prior to vaccination, look for any visits for shoulder or upper arm problems, symptoms, or injuries. Do not worry about any injuries or symptoms to the elbow, forearm, wrist, or hand.

9. Any prior shoulder/upper arm symptoms found?

◯ Yes
◯ No

9-1). Does the side of the shoulder symptoms match the interested laterality?

◯ Yes
◯ No
◯ Unknown

9-2). If the answer to 9-1) is Unknown, state your reasoning.

10. Visit date of most recent visit for shoulder/upper arm symptoms prior to vaccination

____

D. NEXT MEDICAL ENCOUNTER AFTER VACCINATION

We are interested in the first medical encounter that happened on a date within [1, 30] days of vaccination, even if it is not related to the shoulder/arm or vaccination.

11. Is there an encounter within 30 days of vaccination?

◯ Yes
◯ No

12. Date of next medical encounter

____

13. Type of encounter

◯ Outpatient (primary care and specialty care office visit)
◯ Urgent care
◯ Emergency room
◯ Hospital inpatient
◯ Phone call
◯ Email
◯ Other (specify)

13-1). Specify other.

____

14. Provider type

◯ Primary Care
◯ ER
◯ Urgent Care
◯ Inpatient Shoulder specialist
◯

© 2026 Zheng C et al. *JAMA Internal Medicine*.

Non-shoulder specialist Other (specify)

14-1). If other, specify.

____

15. What was the chief complaint of this visit?

____

16. Were any shoulder/upper arm symptoms documented as part of this encounter?

◯ Yes
◯ No

16-1). Does the side of the shoulder symptoms match the interested laterality?

◯ Yes
◯ No
◯ Unknown

16-2). If the answer to 16-1) is Unknown, state your reasoning.

____

E. FIRST SHOULDER/UPPER ARM VISIT WITHIN 30 DAYS POST-VACCINATION

Look for the first clinic visit for shoulder/upper arm symptoms matches with the 'interested laterality', within 30 days of vaccination. If the visit is clearly NOT for the shoulder of interest, go to the first visit that is for the shoulder of interest/both shoulders/laterality is unclear.

If the answers to the previous questions (Q16 and Q16-1) are Yes, answer these questions based on that visit. If the answer was No, look for the next clinic visit that involves shoulder/upper arm symptoms and answer the questions based on that visit.

17. Is there a shoulder/upper arm visit within 30 days of the vaccination date?

◯ Yes
◯ No

18. Date of the first shoulder/upper arm visit.
(If there are no shoulder visits, please enter '09/09/9999'. ______________________________

19. Type of encounter

◯ Outpatient (primary care and specialty care office visit)
◯ Urgent care
◯ Emergency room
◯ Hospital inpatient
◯ Other (specify)

19-1). Specify other.

____

20. Type of provider

◯ Orthopedics
◯ Rheumatologist
◯ Physical Medicine & Rehabilitation
◯ Sports Medicine
◯ Primary Care Physician

© 2026 Zheng C et al. *JAMA Internal Medicine*.

◯ Physical Therapy
◯ Occupational Therapy
◯ Other (specify)

20-1). Specify other provider type. ____________________

21. When did the shoulder or arm symptoms start?
(Leave blank if symptom duration or onset date not discussed at this visit.)

____

Please be as specific as possible. If a date was given, provide it. If a duration is given (e.g., "two weeks ago") count backward that amount of time from the visit date and use that as the start date, even if the duration given was inexact (e.g., "about two weeks ago"). If a range is given (e.g., "one to two weeks ago"), use the earliest possible start date (in that case, two weeks prior to the appointment). Use the calendar box to select a date and write the exact statement regarding the start date or symptom duration in the text box.

22. Is this date exact or an estimation?

◯ Exact
◯ Estimate

(Select "estimate" if the duration of symptoms was not discussed.)

23. Is this a pre-existing condition?

◯ Yes
◯ No
◯ Possible
◯ Unknown

24. Were the pre-existing shoulder/arm symptoms noted to recur or worsen following vaccination?

◯ Yes
◯ No
◯ Possible
◯ Unknown

If 'No', please STOP here and go to Q48.

25. Did the recurrence or worsening of symptoms occur within 30 days of vaccination?

◯ Yes
◯ No
◯ Unknown

If 'No', finish this question then go to Q48.

Give the statement regarding the description of the symptoms, start date or symptom duration as it is in the chart. If duration is not discussed, enter that here as well.

____

Please save the full visit note (unredacted) to the S: drive as part of a case packet.

26. Check all the symptoms that were reported.

☐ Atrophy
☐ Impingement
☐
☐
☐
☐
☐

© 2026 Zheng C et al. *JAMA Internal Medicine*.

Numbness
Pain/Soreness
Radiating pain (from a non-shoulder location)
Reduced range of motion (ROM)
Stiffness
Swelling
Tingling
Weakness

26-1). Specify other.

____

27. Was a specific injury or action, other than vaccination, mentioned as the cause of the shoulder/upper arm symptoms?

- ○ Yes
- ○ No

28. Were the shoulder/upper arm symptoms attributed to the vaccine by the patient or provider?

- ○ Yes
- ○ No

29. Please copy and paste the relevant statement from the chart note for any cause of the symptoms AND
save the full visit note (unredacted) to S: drive as part of a case packet.

30. Was vaccination mentioned at all in the visit note?

- ○ Yes
- ○ No

31. Was the vaccine type specified in the statement?

- ○ Yes
- ○ No

31-1). Does the vaccine type state match the one listed in Q5?

- ○ Yes
- ○ No

F. FIRST SHOULDER/UPPER ARM VISIT WITHIN 31-180 DAYS POST-VACCINATION

Look for the first clinic visit for shoulder/upper arm symptoms, within 31-180 days of vaccination. If the visit is clearly NOT for the shoulder of interest, go to the first visit that is for the shoulder of interest/both shoulders/laterality is unclear.

32. Is there a visit related to the shoulder/arm of interest within 31-180 days following vaccination? If 'No', go to Section G.

- ○ Yes
- ○ No

33. Date of visit:

© 2026 Zheng C et al. *JAMA Internal Medicine*.

34. Type of encounter:

- ○ Outpatient (primary care and specialty care office visit)
- ○ Urgent care
- ○ Emergency room
- ○ Hospital inpatient
- ○ Other (specify)

34-1). Specify other.

35. Type of provider:

- ○ Orthopedics
- ○ Rheumatologist
- ○ Physical Medicine & Rehabilitation
- ○ Sports Medicine
- ○ Primary Care Physician (e.g., internal medicine, family medicine, pediatrics)
- ○ Physical Therapy
- ○ Occupational Therapy
- ○ Other (specify)

35-1). Specify other provider types.

36. Which shoulder was discussed at the encounter?

- ○ Right
- ○ Left
- ○ Both
- ○ Unknown

37. Did the patient note that his/her symptoms had resolved/relieved?

- ○ Resolved
- ○ Improved
- ○ No resolution/improvement
- ○ Other (e.g., some symptoms improved but other symptoms still present)
- ○ Unknown

38. Was a specific injury or action, other than vaccination, mentioned as the cause of the shoulder/upper arm symptoms?

- ○ Yes
- ○ No

39. Were the shoulder/upper arm symptoms attributed to the vaccine by the patient or provider?

- ○ Yes
- ○ No

40. Please copy and paste the relevant statement from the chart note for any cause of the

© 2026 Zheng C et al. *JAMA Internal Medicine*.

symptoms and save the full visit note (unredacted) to the S: drive as part of the case packet.

41. Was vaccination mentioned at all in the visit note?

◯ Yes
◯ No

42. Was the vaccine type specified in the statement?

◯ Yes
◯ No

42-1). If the above question is "Yes", specify vaccine type.

____

42-2). Does the vaccine type stated match the one listed in Q5?

◯ Yes
◯ No

G. Case Definition

A SIRVA case is a shoulder injury occurring in the same arm in which a vaccine was injected within the first 7 days following vaccination and lasting more than 30 days following vaccination, with vaccination as one of the possible causes of the shoulder injury.

Please use all available records in -12 to +6 months from the index date to answer the questions in this section.

43. In your review, which shoulder/arm was identified?

◯ Right
◯ Left
◯ Both
◯ Unknown

44. Did the shoulder injury occur in the same arm in which a vaccine was injected?

◯ Yes
◯ No
◯ Unknown

44-1). Please copy and paste the relevant statement from the chart notes on the shoulder injury.

____

45. Did the symptoms of shoulder injury begin within the first 7 days following vaccination? (Please be sure to use the most complete and reliable information to answer this question when there are discrepancies with more than one source.)

◯ Yes
◯ No
◯ No, but increased severity of pre-existing symptoms in the first 30 days
◯ Possible
◯ Unknown

45-1). Please copy and paste the relevant statement from the chart notes on the shoulder injury symptom onset.

____

46. Did the shoulder symptoms persist for more than 30 days?

◯ Yes
◯ No
◯ Possible
◯ Unsure

© 2026 Zheng C et al. *JAMA Internal Medicine*.

46-1). Please copy and paste the relevant statement from the chart notes on the shoulder injury symptom duration.

____

47. What was the cause of the shoulder injury? (Please select as many causes as documented.)
- ☐ Vaccine
- ☐ Incident (e.g. fall, auto accident)
- ☐ Exercise (e.g. exercise, sports)
- ☐ Daily activity (e.g. overuse, lifting a heavy item, work-related injury, side sleeping)
- ☐ Other medical conditions (e.g. arthritis, chest pain radiating to the shoulder)
- ☐ Unknown (e.g. no explicit cause, insidious/aggravating factors- 'worse with exercise')

47-1). Please copy and paste the relevant statement from the chart notes on the cause of shoulder injury (for all of the above responses).

____

48. Does it look like a SIRVA case?
- ◯ Yes
- ◯ No
- ◯ Possible
- ◯ Unsure

49. Please specify the reasoning when you answer Q48:

____

50. Do you have any questions or comments not addressed in the form? Please enter them here.

____

© 2026 Zheng C et al. *JAMA Internal Medicine*.

eFigure 1. Illustrations of Examples of Influenza and COVID-19 Vaccinations Administered Between August 1, 2023, and April 30, 2024

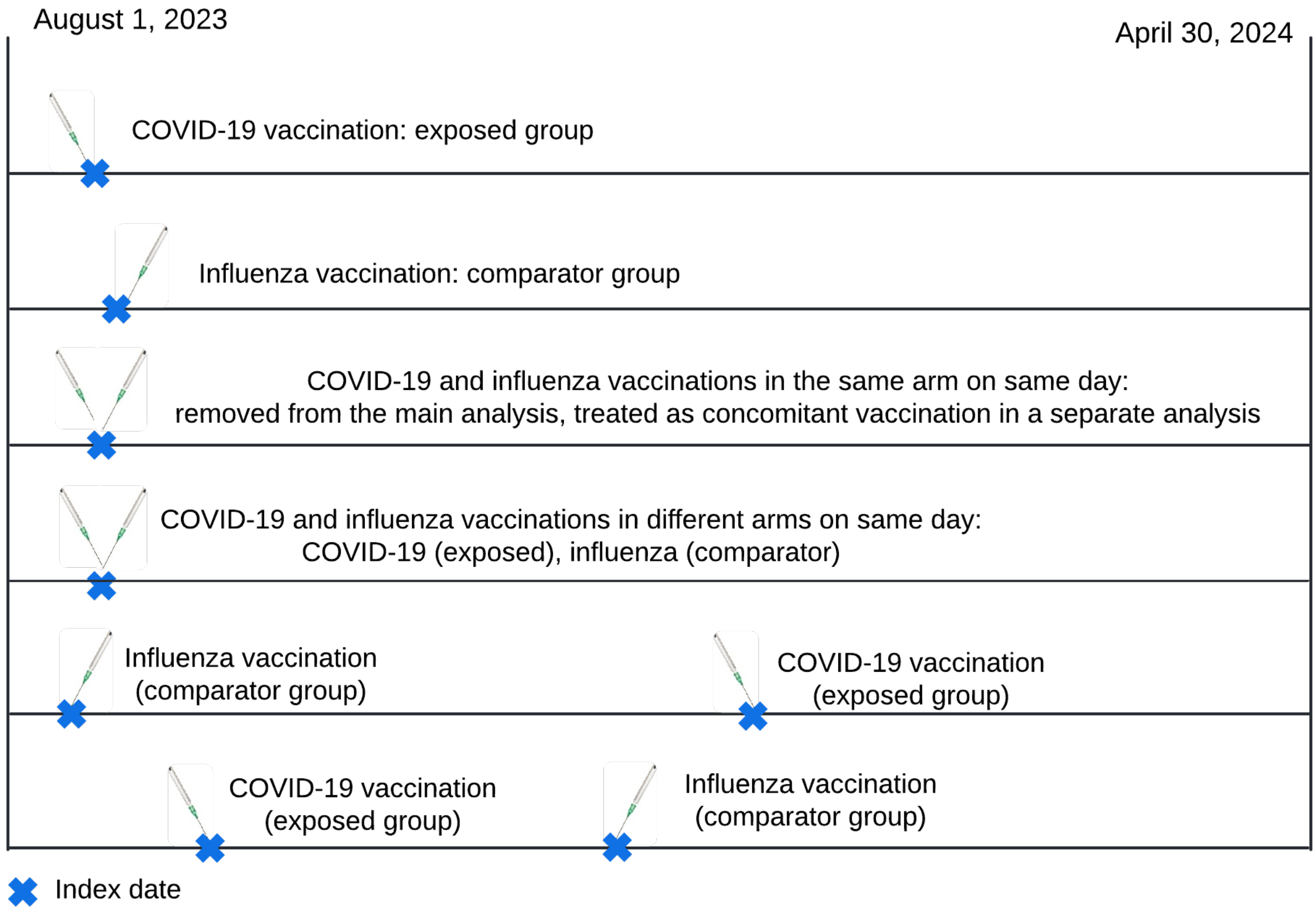

© 2026 Zheng C et al. *JAMA Internal Medicine*.

eFigure 2. Comparison of Absolute Standardized Difference Before and After IPTW Among Influenza-Vaccinated and COVID-19-Vaccinated Individuals, Kaiser Permanente Southern California, August 1, 2023-April 30, 2024

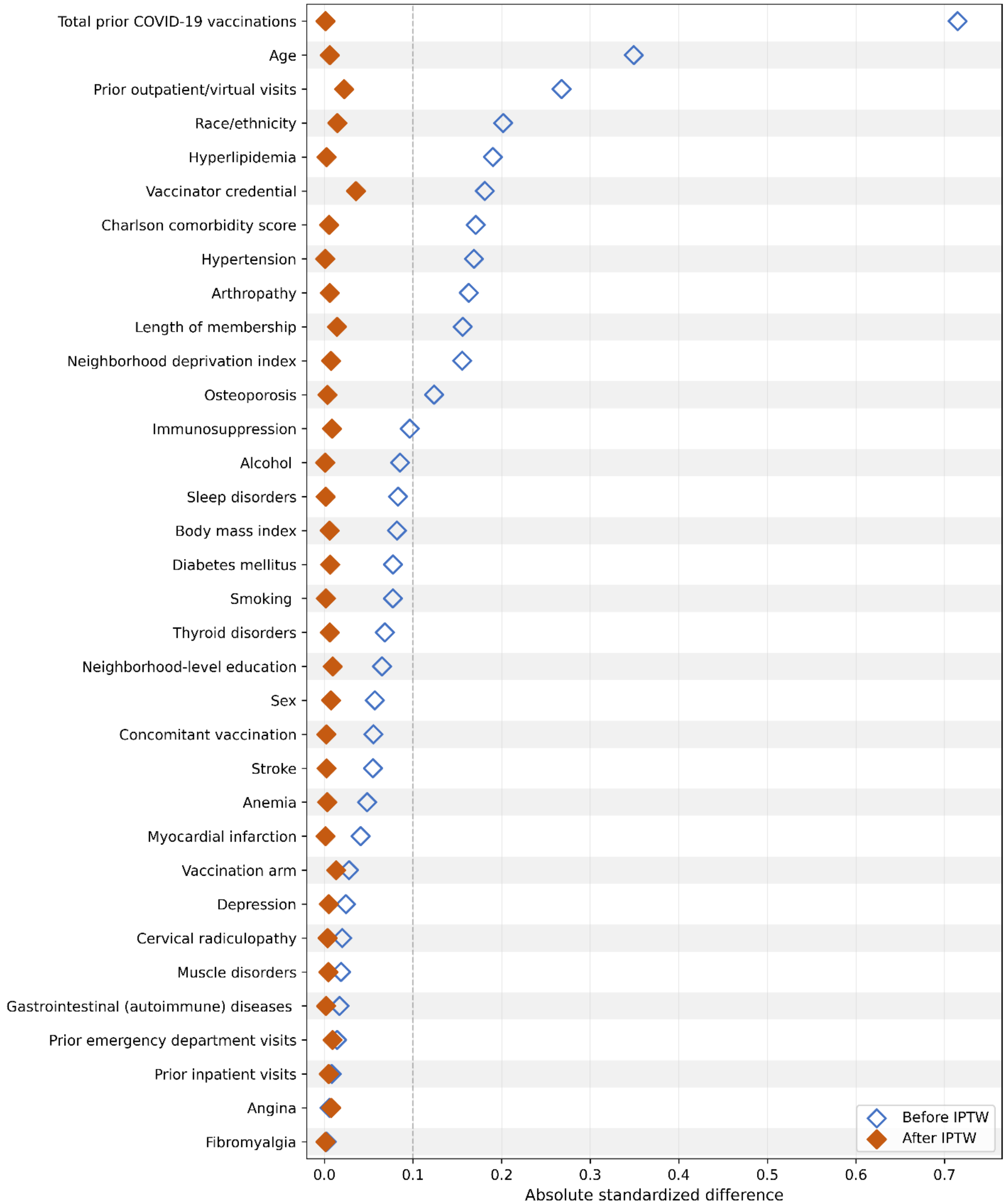

Abbreviation: IPTW, inverse probability of treatment weighting.

Concomitant vaccination: COVID-19 or influenza vaccinations co-administered in the same arm and on the same day with another vaccine other than COVID-19 or influenza.

© 2026 Zheng C et al. *JAMA Internal Medicine*.

eTable 1. ICD-10-CM Code Groups for Identifying Presumptive Shoulder Injury Cases

Code group	Description
ICD-10-CM code group A	Shoulder disorder diagnoses reported in the SIRVA literature
ICD-10-CM code group B	Other shoulder disorder diagnoses not previously reported in the SIRVA literature
ICD-10-CM code group C	Shoulder symptom codes
ICD-10-CM code group D	Shoulder injury codes (ICD-10-CM chapter 19: Injury, poisoning and certain other consequences of external causes)

ICD-10-CM = International Classification of Diseases, 10th Revision, Clinical Modification.

The complete list of ICD-10-CM codes for each code group is listed below.

© 2026 Zheng C et al. *JAMA Internal Medicine*.

eTable 2. ICD-10-CM Code Group A (Shoulder Disorder Codes From SIRVA Literature)

Code	Diagnosis	Laterality		
		Left	Right	Unspecified
**Shoulder bursitis diagnosis codes**				
M75.5X	Bursitis of Shoulder	M75.52	M75.51	M75.50
M71.31	Other bursal cyst, shoulder	M71.312	M71.311	M71.319
M71.81	Other specified bursopathies, shoulder	M71.812	M71.811	M71.819
**Shoulder diagnosis codes (not bursitis)**				
M02.21X	Postimmunization arthropathy	M02.212	M02.211	M02.219
M13.11X	Monoarthritis, not elsewhere classified, shoulder	M13.112	M13.111	M13.119
M13.81X	Other specified arthritis, shoulder	M13.812	M13.811	M13.819
M25.41X	Effusion of shoulder	M25.412	M25.411	M25.419
M65.11X	Other infective tenosynovitis, shoulder	M65.112	M65.112	M65.119
M65.81X	Other synovitis and tenosynovitis, shoulder	M65.812	M65.811	M65.819
M67.31	Transient synovitis, shoulder	M67.312	M67.311	M67.319
M67.81	Other specified disorders of synovium and tendon, shoulder	M67.812, M67.814	M67.811, M67.813	M67.819
M67.91	Unspecified disorder of synovium and tendon, shoulder	M67.912	M67.911	M67.919
M67.92	Unspecified disorder of synovium and tendon, upper arm	M67.922	M67.921	M67.929
M75.0X	Adhesive capsulitis/frozen shoulder	M75.02	M75.01	M75.00
M75.1XX	Tears of rotator cuff	M75.102, M75.112, M75.122	M75.101, M75.111, M75.121	M75.100, M75.110, M75.120
M75.2X	Bicipital tendinitis	M75.22	M75.21	M75.20
M75.3X	Calcific tendinitis of shoulder	M75.32	M75.31	M75.30
S46.00X	Unspecified injury of muscle(s) and tendon(s) of rotator cuff	S46.002*	S46.001*	S46.009*
S46.09X	Other injury of muscle(s) and tendon(s) of rotator cuff	S46.092*	S46.091*	S46.099*
S40.01X	Contusion of shoulder	S40.012*	S40.011*	S40.019*
S46.01X	Strain of muscle(s) and tendon(s) of the rotator cuff	S46.012*	S46.011*	S46.019*

© 2026 Zheng C et al. *JAMA Internal Medicine*.

eTable 3. ICD-10-CM Code Group B (Shoulder Disorder Codes not Previously Reported in the SIRVA Literature)

		Laterality		
**Code**	**Diagnosis**	**Left**	**Right**	**Unspecified**
M24.11	Other articular cartilage disorders, shoulder	M24.112	M24.111	M24.119
M24.21	Disorder of ligament, shoulder	M24.212	M24.211	M24.219
M24.51	Contracture, shoulder	M24.512	M24.511	M24.519
M24.61	Ankylosis, shoulder	M24.612	M24.611	M24.619
M24.81	Other specific joint derangements of shoulder, not elsewhere classified	M24.812	M24.811	M24.819
M25.01	Hemarthrosis, shoulder	M25.012	M25.011	M25.019
M25.11	Fistula, shoulder	M25.112	M25.111	M25.119
M25.21	Flail joint, shoulder	M25.212	M25.211	M25.219
M25.31	Other instability, shoulder	M25.312	M25.311	M25.319
M25.71	Osteophyte, shoulder	M25.712	M25.711	M25.719
M25.81	Other specified joint disorders, shoulder	M25.812	M25.811	M25.819
M60.21	Foreign body granuloma of soft tissue, not elsewhere classified, shoulder	M60.212	M60.211	M60.219
M60.22	Foreign body granuloma of soft tissue, not elsewhere classified, upper arm	M60.222	M60.221	M60.229
M60.81	Other myositis shoulder	M60.812	M60.811	M60.819
M60.82	Other myositis upper arm	M60.822	M60.821	M60.829
M61.01	Myositis ossificans traumatica, shoulder	M61.012	M61.011	M61.019
M61.02	Myositis ossificans traumatica, upper arm	M61.022	M61.021	M61.029
M61.41	Other calcification of muscle, shoulder	M61.412	M61.411	M61.419
M61.42	Other calcification of muscle, upper arm	M61.422	M61.421	M61.429
M61.51	Other ossification of muscle, shoulder	M61.512	M61.511	M61.519
M61.52	Other ossification of muscle, upper arm	M61.522	M61.521	M61.529

© 2026 Zheng C et al. *JAMA Internal Medicine*.

M62.01	Separation of muscle (nontraumatic), shoulder	M62.012	M62.011	M62.019
M62.02	Separation of muscle (nontraumatic), upper arm	M62.022	M62.021	M62.029
M62.11	Other rupture of muscle (nontraumatic), shoulder	M62.112	M62.111	M62.119
M62.12	Other rupture of muscle (nontraumatic), upper arm	M62.122	M62.121	M62.129
M62.21	Nontraumatic ischemic infarction of muscle, shoulder	M62.212	M62.211	M62.219
M62.22	Nontraumatic ischemic infarction of muscle, upper arm	M62.222	M62.221	M62.229
M62.41	Contracture of muscle, shoulder	M62.412	M62.411	M62.419
M62.41	Contracture of muscle, upper arm	M62.422	M62.421	M62.429
M62.51	Muscle wasting and atrophy, not elsewhere classified, shoulder	M62.512	M62.511	M62.519
M62.52	Muscle wasting and atrophy, not elsewhere classified, upper arm	M62.522	M62.521	M62.529
M66.11	Rupture of synovium, shoulder	M66.112	M66.111	M66.119
M66.21	Spontaneous rupture of extensor tendons, shoulder	M66.212	M66.211	M66.219
M66.22	Spontaneous rupture of extensor tendons, upper arm	M66.222	M66.221	M66.229
M66.31	Spontaneous rupture of flexor tendons, shoulder	M66.312	M66.311	M66.319
M66.32	Spontaneous rupture of flexor tendons, upper arm	M66.322	M66.321	M66.329
M66.81	Spontaneous rupture of other tendons, shoulder	M66.812	M66.811	M66.819
M66.82	Spontaneous rupture of other tendons, upper arm	M66.822	M66.821	M66.829
M67.21	Synovial hypertrophy, not elsewhere classified, shoulder	M67.212	M67.211	M67.219
M67.22	Synovial hypertrophy, not elsewhere classified, upper arm	M67.222	M67.221	M67.229
M67.41	Ganglion, shoulder	M67.412	M67.411	M67.419
M70.81	Other soft tissue disorders related to use, overuse and pressure of shoulder	M70.812	M70.811	M70.819
M70.91	Unspecified soft tissue disorder related to use, overuse and pressure of shoulder	M70.912	M70.911	M70.919
M75.4	Impingement syndrome of shoulder	M75.42	M75.41	M75.40

© 2026 Zheng C et al. *JAMA Internal Medicine*.

M75.8	Other shoulder lesions	M75.82	M75.81	M75.80
M75.9	Shoulder lesion, unspecified	M75.92	M75.91	M75.90
M79.A1	Nontraumatic compartment syndrome of upper extremity	M79.A12	M79.A11	M79.A19
**<u>Other osteopathies</u>**				
M89.01	Algoneurodystrophy, shoulder	M89.012	M89.011	M89.019
M89.02	Algoneurodystrophy, upper arm	M89.022	M89.021	M89.029
M89.71	Major osseous defect, shoulder region	M89.712	M89.711	M89.719
M89.72	Major osseous defect, humerus	M89.722	M89.721	M89.729
M89.8X1	Other specified disorders of bone, shoulder			M89.8X1
M89.8X2	Other specified disorders of bone, upper arm			M89.8X2
**<u>Chondropathies</u>**				
M94.21	Chondromalacia, shoulder	M94.212	M94.211	M94.219
M94.8X1	Other specified disorders of cartilage, shoulder			M94.8X1
M94.8X2	Other specified disorders of cartilage, upper arm			M94.8X2

© 2026 Zheng C et al. *JAMA Internal Medicine*.

eTable 4. ICD-10-CM Code Group C (Shoulder Symptom Codes)

		Laterality		
**Code**	**Diagnosis**	**Left**	**Right**	**Unspecified**
M25.51	Pain in shoulder	M25.512	M25.511	M25.519
M25.61	Stiffness of shoulder, not elsewhere classified	M25.612	M25.611	M25.619
M79.6	Pain in arm	M79.602	M79.601	M79.609
M79.62	Pain in upper arm	M79.622	M79.621	M79.629

© 2026 Zheng C et al. *JAMA Internal Medicine*.

eTable 5. ICD-10-CM Code Group D (From ICD-10-CM Chapter 19: Injury, Poisoning and Certain Other Consequences of External Causes)

		Laterality		
**Code**	**Diagnosis**	**Left**	**Right**	**Unspecified**
S44	Injury of nerves at shoulder and upper arm level	S44.32X*, S44.8X2*, S44.92X*	S44.31X*, S44.8X1*, S44.91X*	S44.30X*, S44.8X9*, S44.90X*
S46	Injury of muscle, fascia and tendon at shoulder and upper arm level	S46.002*, S46.012*, S46.022*, S46.092*, S46.802*, S46.812*, S46.822*, S46.892*, S46.902*, S46.912*, S46.922*, S46.982*, S46.992*	S46.001*, S46.011*, S46.021* S46.091*, S46.801*, S46.811*, S46.821*, S46.891*, S46.901*, S46.911*, S46.921*, S46.981*, S46.991*	S46.009*, S46.019*, S46.029* S46.099*, S46.809*, S46.819*, S46.829*, S46.899*, S46.909*, S46.919*, S46.929*, S46.989*, S46.999*

© 2026 Zheng C et al. *JAMA Internal Medicine*.

eTable 6. Assessment Interval Prior to the Index Date for Comorbidities

Comorbidity	Assessment interval prior to index date[a]	ICD-10-CM code[b]
**Anemia**	1 year	
Iron deficiency anemia		D50
Other anemias		D51, D52, D53, D55, D56, D57, D58, D59, D60, D61.1, D61.3, D61.8, D62, D63, D64
Anemia based on lab data	Lab test data prior to and closest to the index date.	Hgb <13 g/dL for males, Hgb <12 g/dL for females.
**Angina (chest pain)**	4 years	I20.1, I20.8, I20.9, I25.111, I25.118, I25.119, I25.701, I25.708, I25.709, I25.711, I25.718, I25.719, I25.721, I25.728, I25.729, I25.731, I25.738, I25.739, I25.751, I25.758, I25.759, I25.761, I25.768, I25.769, I25.791, I25.798, I25.799, R07.1, R07.2, R07.8, R07.9
**Arthropathy**	From 10/1/2015	
Osteoarthritis		M15, M16, M17, M18, M19
Rheumatoid Arthritis		M05, M06, M08
Gout		M10, M1A
Other		M00, M01, M02, M11, M12, M13, M14
**Cervical radiculopathy**	4 years	M54.12, M50.12, M47.22
**Depression**	4 years	F30, F31, F32, F33, F34, F39
**Diabetes**	4 years	E10, E11
**Fibromyalgia**	4 years	M79.7
**Immune-mediated gastrointestinal disorders**	4 years	K90.0, K50, K51
**Hyperlipidemia**	4 years	E78.0, E78.1, E78.2, E78.3, E78.4, E78.5

© 2026 Zheng C et al. *JAMA Internal Medicine*.

**Hypertension**	4 years	I10, I11, I12, I13, I15, I16.9, I1A
**Immunosuppression**		
Solid malignancy	4 years	C00–C80, C7A, C7B, D3A, Z51.0, and Z51.1
Hematologic malignancy	4 years	C81–C86, C88, C90–C96, D46, D61.0, D61.2, D61.9, D70.0, and D71
Rheumatologic or inflammatory disorder	From 10/1/2015	D86, E85 [except E85.0], G35, J67.9, L40.59, L93.0, L93.2, L94, M07, M30, M31.3, M31.5, M32–M34, M35.3, M35.8, M35.9, M46, and T78.40
Other intrinsic immune condition or immunodeficiency	From 10/1/2015	D27.9, D61.09, D72.89, D80, D81 [except D81.3], D82–D84, D89 [except D89.2], K70.3, K70.4, K72, K74.3–K74.6 [except K74.60 and K74.69], N04, and R18
Organ or stem cell transplant	From 10/1/2015	T86 [except T86.82–T86.84, T86.89, and T86.9], D47.Z1, Z48.2, Z94, and Z98.85
Human immunodeficiency virus	From 10/1/2015	B20, R75, Z21, B97.35, O98.7
Spleen-related conditions	From 10/1/2015	D73.0, D73.1, Z90.81
Long-term use of immunosuppressants or steroids	From 1/1/2023[c]	Z79.62, Z79.63, Z79.64, Z79.5
**Thyroid disorders**	4 years	E03, E04, E05, E06, E07
**Myocardial infarction**	From 10/1/2015	I21.0, I21.1, I21.2, I21.3, I21.4, I21.9, I21.A, I22, I25.2
**Muscle disorders**	4 years	G71, G72, M60, M61, M62, M63
**Osteoporosis**	4 years	M80, M81
**Sleep disorder**	4 years[d]	G47, F51, G25.8, R06.81
**Stroke**	From 10/1/2015	I60, I61, I62, I63, I65, I66, I69, G43.6, G45, G46, R29.7

Because individuals could receive multiple vaccinations during the study period, we assigned person-level characteristics to each vaccination based on the individual's status at the time of that index vaccination. For time-varying covariates (e.g., prior vaccination count), values were updated for each index vaccination an individual received. For time-invariant characteristics (e.g., sex, race/ethnicity), the same value was assigned to all index vaccinations for that individual.

[a] Lookback period before the index vaccination date. For conditions that might have lasting effects, such as arthropathy, myocardial infarction, and stroke, we looked back to 10/1/2015 (the ICD-10-CM implementation date).

[b] Only top-level ICD-10 codes are shown; all corresponding sub-level codes were included.

© 2026 Zheng C et al. *JAMA Internal Medicine*.

[c] These were new ICD-10-CM codes from 1/1/2023.

[d] ≥ 2 instances of diagnostic codes on separate dates. Based on KPSC validation data (https://doi.org/10.5664/jcsm.8160), this algorithm achieved 100% positive predictive value and 99% negative predictive value.

© 2026 Zheng C et al. *JAMA Internal Medicine*.

eTable 7. Types of Influenza and COVID-19 Vaccines in the Eligible, Presumptive, and SIRVA Cohorts, Kaiser Permanente Southern California, August 1, 2023-April 30, 2024

	Eligible (n = 1 901 588)		Presumptive (n = 32 484)		SIRVA (n = 265)	
	n	%	n	%	n	%
**Influenza vaccines**	1 304 974		21 709		155	
SD-IIV4	866 530	66.40	12 347	56.87	86	55.49
HD-IIV4	438 183	33.58	9358	43.11	69	44.52
RIV4	258	0.02	4	0.02	0	0
ccIIV4	3	0	0	0	0	0
**COVID-19 vaccines**	667 566		11 677		123	
Pfizer XBB	667 455	99.98	11 675	99.98	123	100
Novavax XBB	111	0.02	2	0.02	0	0

Abbreviations: HD-IIV4, high-dose inactivated influenza vaccine quadrivalent; SD-IIV4, standard-dose inactivated influenza vaccine quadrivalent; RIV4, recombinant hemagglutinin influenza vaccine quadrivalent; ccIIV4, cell culture-based influenza vaccine quadrivalent.

Simultaneous COVID-19 and influenza vaccinations were counted in both the COVID-19 vaccine group and the influenza vaccine group.

© 2026 Zheng C et al. *JAMA Internal Medicine*.

eTable 8. Agreement Between the Orthopedic Surgeon and Research Associates and Adjudicated Final Classification Among 40 Probable or Possible Cases

Reviewer 1	Reviewer 2	Final Consensus: Positive	Final Consensus: Negative	Total Cases
**Positive**	**Positive**	31	0	31
**Positive**	**Negative**	3	2	5
**Negative**	**Positive**	0	1	1
**Negative**	**Negative**	0	3	3
**Total**		34	6	40

Reviewer 1: orthopedic shoulder surgeon

Reviewer 2: research associates

Inter-rater agreement between the orthopedic shoulder surgeon and research associates was 85% (34/40; 95% CI, 70.2%–94.3%), with a Cohen κ of 0.42 (95% CI, 0–0.85). The wide confidence interval for Cohen κ reflected the small double-reviewed sample and the predominance of positive cases, which resulted in high expected agreement by chance and limited precision in estimating chance-corrected agreement.

© 2026 Zheng C et al. *JAMA Internal Medicine*.

eTable 9. Timing of First and Last Shoulder-Related Encounters During the 180-Day Follow-up Among Confirmed SIRVA Cases, by Vaccination Group

	Days post-vaccination		
	Influenza (n=142)	COVID-19 (n=110)	Simultaneous (n=13)[a]
**First shoulder encounter**			
Min, Max	0, 174	1, 169	24, 133
Mean (SD)	56.2 (41.2)	63.3 (39.5)	58.9 (33)
Median (IQR)	46.0 (27.2-74.5)	51.5 (33-89.8)	50.0 (32-67)
**Last shoulder encounter**			
Min, Max	31, 178	41, 179	50, 172
Mean (SD)	98.7 (48.6)	121.9 (43)	117.5 (44.5)
Median (IQR)	99.5 (54-140.5)	130.5 (83.2-160.8)	122 (77-158)

[a] COVID-19 and influenza vaccines were co-administered in the same arm on the same day.

© 2026 Zheng C et al. *JAMA Internal Medicine*.

eTable 10. Timing of Additional Same-Arm Vaccinations Before or After the SIRVA-Associated Vaccination Among Confirmed SIRVA Cases

Interval from SIRVA-associated vaccination, days	Before (n=72 vaccinations)	After (n=26 vaccinations)
Mean (SD)	103.8 (73.4)	107.6 (65.2)
Median (IQR)	73 (52-178)	104 (49-162)

Among the 265 confirmed SIRVA cases, 81 had at least 1 additional vaccination administered in the same arm during the study period (August 1, 2023, to April 30, 2024) or within 180 days before or after the SIRVA-associated vaccination. There were 72 same-arm vaccination events before the SIRVA-associated vaccination among 63 unique cases and 26 same-arm vaccination events after the SIRVA-associated vaccination among 24 unique cases. Six cases had same-arm vaccinations both before and after the SIRVA-associated vaccination; therefore, the before and after groups were not mutually exclusive. For these 81 cases, manual review confirmed that we correctly identified the SIRVA-associated vaccination.

© 2026 Zheng C et al. *JAMA Internal Medicine*.

eTable 11. Characteristics of Cohorts Who Received COVID-19 and Influenza Vaccination After Weighting, Kaiser Permanente Southern California, August 1, 2023, to April 30, 2024

Characteristic	Vaccination, No. (%)[a]		ASD
	Influenza vaccination *n* = 1 234 022	COVID-19 vaccination *n* = 596 614	
**Age at index date, y**			
Mean (SD)	58.5 (18.1)	58.5 (18.3)	0.000
Median (IQR)	62 (45-72)	62 (45-72)	
18–49	374 706 (30.4)	181 998 (30.5)	0.006
50–64	312 871 (25.4)	152 211 (25.5)	
≥65	546 445 (44.3)	262 405 (44)	
**Sex**			0.007
Female	700 648 (56.8)	336 547 (56.4)	
Male	533 374 (43.2)	260 067 (43.6)	
**Race and ethnicity[b]**			0.014
Hispanic	459 576 (37.2)	223 720 (37.5)	
Asian and Pacific Islander	204 046 (16.5)	100 048 (16.8)	
Black	87 728 (7.1)	42 830 (7.2)	
Multiple, other, and unknown	51 878 (4.2)	25 678 (4.3)	
White	430 795 (34.9)	204 337 (34.2)	
**Body mass index[c]**			
Mean (SD)	29.3 (6.7)	29.3 (6.7)	0.007
Median (IQR)	28.2 (24.6-32.8)	28.3 (24.6-32.8)	
<25	306 610 (24.8)	148 091 (24.8)	0.004
25<30	375 916 (30.5)	181 554 (30.4)	
30<35	242 637 (19.7)	117 631 (19.7)	
≥35	191 037 (15.5)	92 956 (15.6)	
Unknown	117 821 (9.5)	56 382 (9.5)	
**Alcohol history[d]**	318 430 (25.8)	153 743 (25.8)	0.001
**Smoking history[d]**	315 845 (25.6)	153 117 (25.7)	0.002
**Length of membership, y**			0.014
1-5	341 758 (27.7)	168 906 (28.3)	
6-10	271 432 (22)	130 880 (21.9)	
≥11	620 831 (50.3)	296 827 (49.8)	
**Prior outpatient or virtual visits[d]**			0.022
0	18 492 (1.5)	7724 (1.3)	
1-4	337 004 (27.3)	166 803 (28)	
5-10	386 084 (31.3)	186 547 (31.3)	
≥11	492 442 (39.9)	235 539 (39.5)	
**Prior ED visits[d]**			0.009
0	973 452 (78.9)	472 803 (79.2)	
1	166 679 (13.5)	79 347 (13.3)	
≥2	93 891 (7.6)	44 464 (7.5)	
**Prior inpatient visits[d]**			0.005
0	1 134 499 (91.9)	549 260 (92.1)	
1	72 077 (5.8)	34 201 (5.7)	
≥2	27 446 (2.2)	13 153 (2.2)	

© 2026 Zheng C et al. *JAMA Internal Medicine*.

**Total prior COVID-19 vaccinations**			0.001
0-1	41 532 (3.4)	19 996 (3.4)	
2-3	427 534 (34.6)	206 984 (34.7)	
4-5	677 298 (54.9)	327 283 (54.9)	
≥6	87 658 (7.1)	42 351 (7.1)	
**Vaccinator credential**			0.036
Licensed vocational nurse	986 991 (80)	475 287 (79.7)	
Medical assistant	133 154 (10.8)	69 867 (11.7)	
Registered nurse	100 507 (8.1)	44 920 (7.5)	
Student	9251 (0.7)	4422 (0.7)	
Other credentials	4119 (0.3)	2118 (0.4)	
**Vaccination arm (left)**	892 049 (72.3)	404 967 (67.9)	0.013
**Simultaneous administered with non–COVID-19, noninfluenza vaccination**[e]	46 713 (3.8)	16 726 (2.8)	0.002
**Charlson comorbidity score**[f]			0.005
0	630 141 (51.1)	304 775 (51.1)	
1	178 469 (14.5)	85 248 (14.3)	
≥2	425 412 (34.5)	206 591 (34.6)	
**Comorbidities**[g]			
Anemia	180 964 (14.7)	86 833 (14.6)	0.003
Angina	280 079 (22.7)	133 509 (22.4)	0.008
Arthropathy	425 077 (34.4)	203 770 (34.2)	0.006
Cervical radiculopathy	59 138 (4.8)	28 143 (4.7)	0.004
Depression	250 017 (20.3)	122 021 (20.5)	0.005
Diabetes	305 567 (24.8)	149 368 (25)	0.006
Fibromyalgia	20 395 (1.7)	9768 (1.6)	0.001
Immune-mediated gastrointestinal disorders	13 778 (1.1)	6558 (1.1)	0.002
Hyperlipidemia	731 269 (59.3)	352 830 (59.1)	0.002
Hypertension	571 364 (46.3)	275 994 (46.3)	0.001
Immunosuppression	245 269 (19.9)	116 563 (19.5)	0.009
Thyroid disorders	189 568 (15.4)	90 336 (15.1)	0.006
Myocardial infarction	48 316 (3.9)	23 221 (3.9)	0.001
Muscle disorders	120 918 (9.8)	57 665 (9.7)	0.005
Osteoporosis	138 729 (11.2)	66 446 (11.1)	0.003
Sleep disorders	202 685 (16.4)	97 689 (16.4)	0.001
Stroke	71 401 (5.8)	34 180 (5.7)	0.002
**Neighborhood Deprivation Index, quartile**[h]			0.007
1st	368 952 (29.9)	177 537 (29.8)	
2nd	345 618 (28)	166 513 (27.9)	
3rd	290 909 (23.6)	140 880 (23.6)	
4th	228 105 (18.5)	111 415 (18.7)	
Unknown	438 (0)	269 (0)	
**Neighborhood-level education**			0.009
<50% With a high school education	54 007 (4.4)	26 510 (4.4)	
≥50% With a high school education	1 179 982 (95.6)	570 049 (95.5)	
Unknown	32 (0)	55 (0)	

Abbreviations: ASD, absolute standardized difference; ED, emergency department.

© 2026 Zheng C et al. *JAMA Internal Medicine*.

[a] The unit of analysis is vaccination. Characteristics are presented for individuals at the time of each vaccination. Because individuals could receive multiple vaccinations during the study period, the same person may be represented in both vaccine groups. The numbers of unique individuals in the influenza and COVID-19 vaccination groups were 1 231 959 and 544 577, respectively. The number of individuals who received both influenza and COVID-19 vaccinations was 477 390.

[b] Race and Hispanic ethnicity group (Asian and Pacific Islander, Black, Hispanic, White, or other race and ethnicity [including American Indian or Alaska Native, multiracial, and unknown]) were self-reported and recorded in the electronic health record. All races listed are non-Hispanic.

[c] The most recent data in the year before the index vaccination. BMI is calculated as weight in kilograms divided by height in meters squared.

[d] The most recent data in the year prior to the index vaccination

[e] COVID-19 or influenza vaccination co-administered with another vaccine that was neither COVID-19 nor influenza in the same arm on the same day

[f] Twelve months before the index vaccination

[g] Lookback periods are defined in (eTable 6 in Supplement 1).

[h] The Neighborhood Deprivation Index is a composite measure of socioeconomic status at the census tract level, categorized into quartiles (1st-4th), with the first representing the least deprived and the fourth the most deprived. Quartiles were defined based on the 2022 Kaiser Permanente Southern California enrolled population.

© 2026 Zheng C et al. *JAMA Internal Medicine*.

eTable 12. SIRVA Incidence and Relative Risk After COVID-19 Vaccination Compared to Influenza Vaccination, With Onset Date Within 2 Days After Vaccination, Kaiser Permanente Southern California, August 1, 2023-April 30, 2024

				Relative risk (95% CI)		Absolute risk difference per 10 000 (95% CI)	
**Vaccination**	**n**	**Cases, n**	**Incidence[a]**	**Unadjusted**	**IPTW Adjusted[b]**	**Unadjusted**	**IPTW Adjusted[b]**
Influenza	1 234 022	130	1.05 (0.89–1.25)	Reference	Reference	Reference	Reference
COVID-19	596 614	108	1.81 (1.50–2.19)	1.72 (1.33–2.22)	1.72 (1.22–2.44)	0.76 (0.37–1.14)	0.71 (0.19–1.23)

Abbreviations: CI, confidence interval; IPTW, inverse probability of treatment weighting; SIRVA, shoulder injury related to vaccine administration.

[a] Incidence (95% CI): SIRVA per 10 000 vaccinations

[b] Total prior vaccinations in the 6 months prior to the index date and month of vaccination were removed from the IPTW analysis but directly adjusted in the Poisson regression model as categorical variables.
