# Supplementary material for: Risk of Shoulder Injury Related to Vaccine Administration for COVID-19 and Influenza Vaccinations
Source: JAMA Intern Med. 2026 Nov 4:e265899. Online ahead of print. doi: 10.1001/jamainternmed.2026.5899 (PMC13735196; doi:10.1001/jamainternmed.2026.5899)
Supplement: Supplementary file 2 [file jamainternmed-e265899-s002.pdf]

Data Sharing Statement

Zheng. Risk of Shoulder Injury Related to Vaccine Administration for COVID-19 and Influenza Vaccinations. *JAMA Intern Med*. Published November 04, 2026.
doi:10.1001/jamainternmed.2026.5899

Data
Data available: No
